# Supplementary figures and images for: LINE-1 retrotransposons facilitate horizontal gene transfer into poxviruses
Source: eLife. 2022 Sep 7;11:e63327. doi: 10.7554/eLife.63327 (PMC9578709; doi:10.7554/eLife.63327)

M

RK13

2 pmCherry-E3L

3 RK13-mCherry-E3L

4 HGT3

M

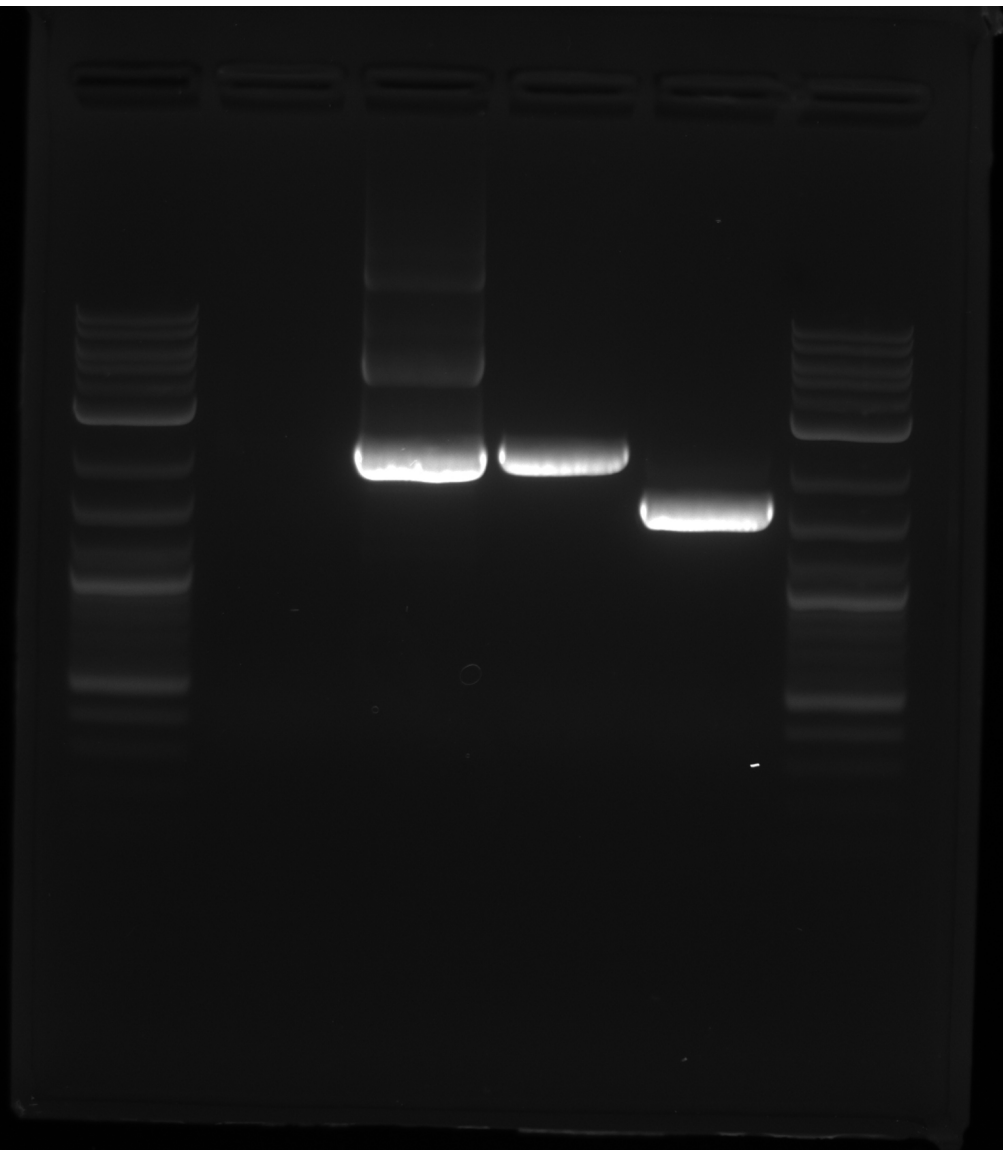

Supplement: Figure 1—figure supplement 1—source data 1. [file elife-63327-fig1-figsupp1-data1.pdf]

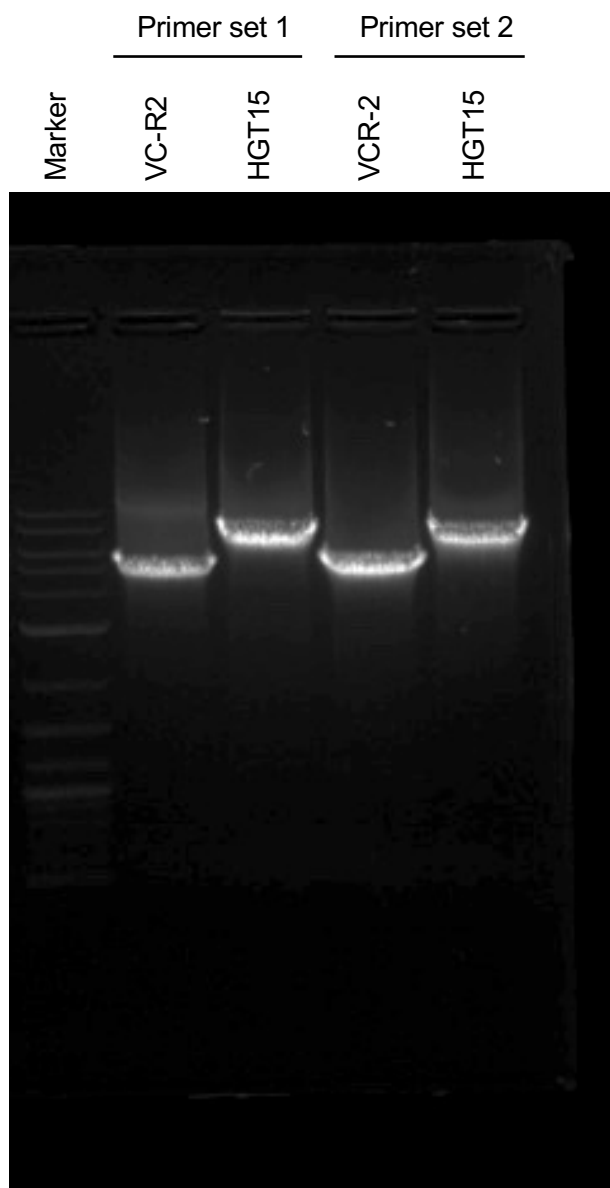

Supplement: Figure 1—figure supplement 4—source data 1. [file elife-63327-fig1-figsupp4-data1.pdf]

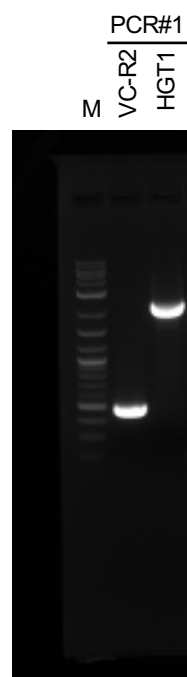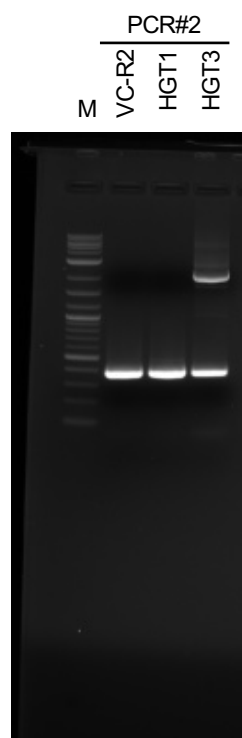

Supplement: Figure 3—source data 5. [file elife-63327-fig3-data5.pdf]

| VC-R2 |    | R1 |   |    | R2 |   |    | R3 |   |    |
|-------|----|----|---|----|----|---|----|----|---|----|
| M     | P0 | 2  | 9 | 17 | 2  | 9 | 17 | 2  | 9 | 17 |

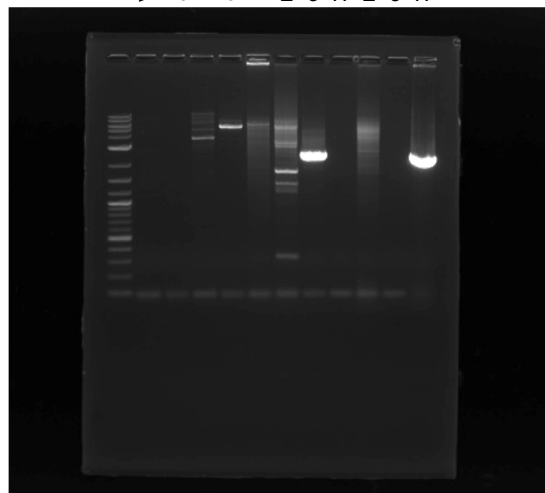

Supplement: Figure 5—source data 1. [file elife-63327-fig5-data1.pdf]
