# Supplementary material for: LINE-1 retrotransposons facilitate horizontal gene transfer into poxviruses
Source: eLife. 2022 Sep 7;11:e63327. doi: 10.7554/eLife.63327 (PMC9578709; doi:10.7554/eLife.63327)
Supplement: Supplementary file 1. [file elife-63327-supp1.docx]

| Clones | Target Site Duplication (TSD) |
| --- | --- |
| HGT1  15 bp TSD | 5’-AGAATAAAGAATCTA-3’  3’-TCTTATTTCTTAGAT-5’ |
| HGT2-15bp  15 bp TSD | 5’-AAGAGTGCCATAACT-3’  3’-TTCTCACGGTATTGA-5’ |
| HGT3  18 bp TSD | 5’-AAGAATGATTACTAACGC-3’  3’-TTCTTACTAATGATTGCG-5’ |
| HGT4  17 bp TSD | 5’-AAAAATTACGATATTCG-3’  3’-TTTTTAATGCTATAAGC-5’ |
| HGT5  16 bp TSD | 5’-AGAAAATTATGTTATG-3’  3’-TCTTTTAATACAATAC-5’ |
| HGT6  18 bp TSD | 5’-AAGAATTTACCTAGTAGC-3’  3’-TTCTTAAATGGATCATCG-5’ |
| HGT7  15 bp TSD | 5’-AAAAAACTTAACTCG-3’  3’-TTTTTTGAATTGAGC-5’ |
| HGT8  15 bp TSD | 5’-AAAATCTGTAGGAGG-3’  3’-TTTTAGACATCCTCC-5’ |
| HGT9  12 bp TSD | 5’-AATCGTTGGAGG-3’  3’-TTAGCAACCTCC-5’ |
| HGT10  14 bp TSD | 5’-AATAGATTCCTTTT-3’  3’-TTATCTAAGGAAAA-5’ |
| HGT12  19 bp TSD | 5’-AATAAAAAAAGTATTTTTT-3’  3’-TTATTTTTTTCATAAAAAA-5’ |
| HGT13  17 bp TSD | 5’-AAGAATCCTATAGGTGG-3’  3’-TTCTTAGGATATCCACC-5’ |
| HGT14  16 bp TSD | 5’-AAGAGTGGATTCTTCT-3’  3’-TTCTCACCTAAGAAGA-5’ |
| HGT15  13 bp TSD | 5’-AATAACTAGCTTA-3’  3’-TTATTGATCGAAT-5’ |
| HGT16  15 bp TSD | 5’-AGGATATGCTTTCAT-3’  3’-TCCTATACGAAAGTA-5’ |
| HGT17  15 bp TSD | 5’-AAATTATTAGAATCA-3’  3’-TTTAATAATCTTAGT-5’ |
| HGT18  17 bp TSD | 5’-AAAAAGGTTTACATTCT-3’  3’-TTTTTCCAAATGTAAGA-5’ |
| HGT19  16 bp TSD | 5’-ATAAAGTGCACGTTTA-3’  3’-TATTTCACGTGCAAAT-5’ |
| HGT20  20 bp TSD | 5’-AAAAAAATATATTATTTTTA-3’  3’-TTTTTTTATATAATAAAAAT-5’ |

**Supplementary File 1. Target site duplications identified in HGT viruses.**
