# Supplementary material for: LINE-1 retrotransposons facilitate horizontal gene transfer into poxviruses
Source: eLife. 2022 Sep 7;11:e63327. doi: 10.7554/eLife.63327 (PMC9578709; doi:10.7554/eLife.63327)
Supplement: Supplementary file 2. [file elife-63327-supp2.docx]

|  | Forward primer (5’-3’) | Reverse primer (5’-3’) |
| --- | --- | --- |
| HGT1 | JR70 vvH4L-1F (H4L):  CTGTGATAGTCGATACGTTATAAAGG | JR73 vvH5R-2F (H5R)  GTTTGGTTTTGGGCTTAGTAGATGG |
| HGT2 | JR75 VV-I8R-1F (I8R)  GGGATTTAAGGTACTAGATGGATCTCC | JR74 VV-I8R-1R (I8R)  GCCGTTCCCTGTCATCCTCTAACG |
| HGT3 | JR35-HGT-H4L-2F (H4L)  CTCGCAAATTCTAGTCTTAACC | JR34-HGT-H4L-2R (H4L)  GAGAGGTCTGTTCTTTCTTCG |
| HGT4 | JR86-G1L-1F (G1L)  GGAATAATCTCCGGACATGCTGG | JR87-G1L-1R (G1L)  CGGTGGGAGAATAGACATGATAG |
| HGT5 | JR117-I4L-4F (I4L)  GATGTTGTGTAGTACAAGTGGC | HGT-E3L-1F (as reverse primer from E3L)  TGGCAGTAGATAAACTTCTTGGTTACG |
|  | HGT-E3L-1R (as forward primer from E3L)  AGCCTCACACACAATCTCTGCG | JR114-I5L-2R (I5L)  CCGTAACGATTTTCAAATATATAGGACTC |
| HGT6 | JR118-D1R-1F (D1R)  GGAGGCAAGGTATTAATCACTACC | JR119-D1R-1R (D1R)  CGTTAAACACTCTGACTATATCGTTC |
| HGT7 | JR165-HGT7-A40-1F (A40R)  GGGAGGAAGGACGTAATAC | JR166-HGT7-A40-1R (A40R)  CTAACCGAAGTAGTGGTATG |
| HGT8 | JR130-A9L-2F (A9L)  GGACTGGAGTTAGAATTTATAGAC | JR129-HGT-A10L-1R (A10L)  GACTAACGAAATCACAGATATG |
| HGT9 | JR131-D9R-1F (D9R)  CGTTCCAGGCGATATTATCTC | JR132-D10R-1R (D10R)  GACTTAGCTAGTCGTCTATTATAC |
| HGT10 | JR131-D9R-1F (D9R)  CGTTCCAGGCGATATTATCTC | JR132-D10R-1R (D10R)  GACTTAGCTAGTCGTCTATTATAC |
| HGT11 | JR190-B4R-1F (B4R)  GAGACGATTACTACTAGTGGATG | JR134-B8R-1R (B8R)  CGACGCATAGTCCCGTAC |
| HGT12 | JR135-A48R-1F (A48R)  CACACGGTTACTGGACC | JR136-A50R-1R (A50R)  GGAAGCAATAGCTTAATGATC |
| HGT13 | JR137-F13L-1F (F13L)  CTATACTAGTCTTAGCTGACC | JR138-F13L-1R (F13L)  CTGGAGGATCTATACATACG |
| HGT14 | JR124-H3L-2F (H3L)  CCGATGATAGACCTCCAG | JR85-H5R-2R (H5R)  GGTGACTAGATCAGATAGTGTTG |
| HGT15 | JR161-HGT15-C19L-1F (C19L)  GTCATCAATGGTGTCCGTTAG | JR162-HGT15-C19L-1R (intergenic space between C19L and C18L)  CATTTACCGGCATCATAAACACG |
| HGT16 | JR186-A26L-1F (A26L)  GATGTTCTGGTTCGACATCC | JR170-A28L-1R (A28L)  CAGGCAGAAGTTGGACC |
| HGT17 | JR171-G5R-1F (G5R)  GCGTTAGAACCACGCAAG | JR187-G7L-1R (G7L)  GATGGAGCTGTGACTAGTC |
| HGT18 | JR173-D6R-1F (D6R)  CGCTTTGTTGTTCGCCTTG | JR174-D6R-1R (D6R)  GAGTTATTGTAGCGAGATAATCC |
| HGT19 | JR242-D11L-1F (D11L)  GGTCTTCGTCTACAGTAGG | HGT-CHR-1R  ATGGCCATGTTATCCTCCTCGC |
|  | HGT-E3L-1F  TGGCAGTAGATAAACTTCTTGGTTACG | JR236-D11L-1R (D11L)  GAATCAAGGCGGTGGC |
| HGT20 | JR175-C2L-1F (C2L)  GATGGTGATCCTGAATGCG | JR178-M1L-1R (M1L)  CTCTGTAGTTAACGGTCATACATG |

**Supplementary File 2. Oligonucleotides used to amplify the integration sites of mCherry-E3L in HGT viruses.**
